# Arg399Gln polymorphism of *XRCC1* gene and risk of colorectal cancer in Kashmir: A case control study

**DOI:** 10.3892/ol.2013.1104

**Published:** 2013-01-03

**Authors:** SANIYA NISSAR, TUFAIL AHMAD LONE, MUJEEB ZAFAR BANDAY, ROOHI RASOOL, NISSAR A. CHOWDRI, FAZL Q. PARRAY, SAFIYA ABDULLAH, AGA SYED SAMEER

**Affiliations:** 1 Departments of Immunology and Molecular Medicine, Sher-I-Kashmir Institute of Medical Sciences, Bemina;; 2 General Surgery, Sher-I-Kashmir Institute of Medical Sciences, Bemina;; 3 Department of Biotechnology, Kashmir University, Hazratbal;; 4 Department of Biochemistry, Medical College, Sher-I-Kashmir Institute of Medical Sciences, Bemina, Srinagar, Kashmir, India

**Keywords:** colorectal cancer, *XRCC1*, polymorphism, Kashmir

## Abstract

The aim of this study was to investigate the role of the *XRCC1* Arg399Gln polymorphism in the susceptibility of a Kashmiri population to colorectal cancer (CRC). We investigated the genotype distribution of the *XRCC1* gene in 130 CRC cases in comparison with that of 150 healthy subjects. There was no direct significant association between the *XRCC1* genotypes and CRC; however, the Arg/Gln genotype was associated with an elevated risk of CRC (OR>1.47) and the Gln/Gln variant genotype was associated with an increased risk of CRC in various clinicopathological parameters. This study suggests that the *XRCC1* polymorphism is associated with an increased risk of CRC.

## Introduction

Colorectal cancer (CRC) is the third most common cancer in males and the second most common cancer in females worldwide (1). In the Kashmir valley, CRC represents the third most common gastrointestinal cancer following esophageal and gastric cancer (2–4).

The Kashmiri population is exposed to a particular set of environmental and dietary risks, including exposure to nitroso compounds, amines and nitrates reported to be present in local foodstuffs, the majority of which have been shown to contain significant irritants and carcinogens (2,3,5,6).

Genetic polymorphisms in DNA repair genes, which lead to amino acid substitution, may influence individual capacity to repair DNA damage, which may be associated with increased genetic instability and carcinogenesis (7). In mammalian cells, four major DNA repair pathways have been identified: base excision repair (BER), nucleotide excision repair (NER), double-strand break repair and mismatch repair (8,9).

The DNA repair gene *XRCC1*, located at 19q13.2, codes for a scaffolding protein physically associated with DNA polymerase β, DNA ligase III, human AP endonuclease, polynucleotide kinase and poly (ADP-ribose) polymerase (8,10,11), which function in a complex to facilitate BER and single-strand break repair processes. The BER pathway mainly removes non-bulky base adducts produced by methylation, oxidation or reduction by ionizing radiation or oxidative damage (12). The *XRCC1* protein is capable of binding directly to both gapped and nicked DNA, as well as to gapped DNA associated with DNA polymerase β, suggesting that this protein may be independently involved in DNA damage recognition (13).

Three polymorphisms occurring at conserved sequences in the *XRCC1* gene have been reported, and amino acid substitutions were detected at codons 194 (Arg>Trp), 280 (Arg>His) and 399 (Arg>Gln) (14). Out of the 3 polymorphisms, the *XRCC1* codon 399 polymorphism has been studied most widely in a number of cancers but with varied results (15–23). While an increased risk has been reported in lung cancer (22) and breast cancer (17,18), there is no definite correlation between *XRCC1* 339 status and the risk of CRC. Abdel-Rahman *et al*(15) were the first to report that the *XRCC1* 399Gln allele, similarly to the *XRCC1* 399Arg/Arg genotype, was associated with an increased risk of developing CRC, particularly amongst young urban residents; however, these results were not reproducible and have not been demonstrated in any other population since. Stern *et al*(24) reported that the 399SNP of the *XRCC1* gene plays a role in modifying the risk of CRC by interactions with fatty acids of the diet. There are also some contradictory reports such as that of Mort *et al*(20), who reported no correlation between CRC risk and polymorphisms in any of the 4 NER genes or in *XRCC1*. Yeh *et al*(21) investigated the involvement of the DNA repair pathway genes in modulating the risk of CRC in a Taiwanese population and found the Arg 399 form to cause an increased cancer risk. Similarly, Skjelbred *et al*(2006)(23) reported a decreased risk of CRC with the Gln399 genotype. More recently, in a study by Wang *et al*(2010) in a South East Indian population (25), the *XRCC1* Gln399 allele was found to significantly increase the risk of rectal cancer. In the present study, we conducted a hospital-based case-controlled investigation to evaluate the potential impact of the *XRCC1* Arg399Gln gene polymorphism on the risk of CRC in a Kashmiri population. We also investigated whether there was a correlation between the clinicopathological variables and the *XRCC1* variant genotype (Gln/Gln), and hence its role in modulating the risk of CRC.

## Materials and methods

### Study population

This study included 130 CRC cases. All patients were recruited from the Department of General Surgery, Sher-I-Kashmir Institute of Medical Sciences, Kashmir, India. Blood samples were collected from 160 ageand gender-matched individuals with no signs of any malignancy to serve as external controls. The mean age in the patient and control groups was 53 years (Table I).

Data on all CRC patients were obtained from personal interviews with patients and/or their guardians, and their medical records. All patients and/or guardians were informed about the study and their willingness to participate was recorded on a predesigned questionnaire (available on request). The collection and use of blood samples (from patients and controls) for this study had been previously approved by the appropriate institutional ethics committee.

### DNA extraction and polymerase chain reaction

DNA extraction was performed using the ammonium precipitation method. Genotyping for the *XRCC1 R399Q* polymorphism was determined using a method described previously (25). The oligonucleotide primers used for the amplification of the target region were: forward F-5′-TTG TGC TTT CTC TGT GTC CA-3′ and reverse R-5′-TCC TCC AGC CTT TTC TGA TA-3′, which generated a 615-bp fragment.

The polymerase chain reaction (PCR) was carried out in a final volume of 20 *μ*l, containing 50 ng genomic DNA template, 1X PCR buffer (Fermentas, Glen Burnie, MD, USA), with 2 mM MgCl_2_, 0.5 *μ*M of each primer (Sigma-Aldrich, Bangalore, India), 50 *μ*M deoxynucleotide triphosphates (dNTPs; Cinnagen, Tehran, Iran) and 0.25 units of DNA polymerase (Invitrogen, Bangalore, India). For PCR amplification, the standard programme was used as follows: one initial denaturation step at 94°C for 7 min, followed by 40 denaturation cycles of 30 sec at 94°C, 30 sec of annealing at 57°C and 30 sec of extension at 72°C for 40 cycles, followed by a final elongation cycle at 72°C for 7 min.

**Figure 1:**
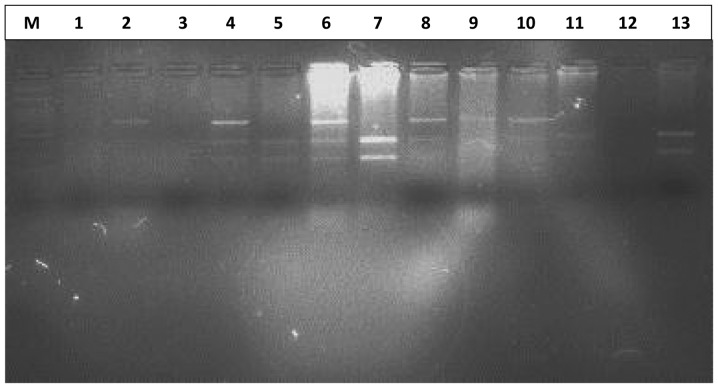
Representative gel of *XRCC1* Arg399Gln polymorphism, showing MspI digested amplicons. The Arg allele is represented by 374 and 241-bp fragments, while the Gln allele is represented by a 615-bp band. Lane M, 100-bp ladder; lanes 2, 9 and 10, homozygous (Gln/Gln) genotype (615 bp); lanes 5, 7, 11 and 13, homozygous (Arg/Arg) genotype (374 and 241 bp); lanes 4, 6 and 8, homozygous (CC) genotype (615, 374 and 241 bp).

The PCR product of *XRCC1* was then digested with 2 units of *MspI* in a reaction mixture of 20 *μ*l for 3 h at 37°C. The Arg allele revealed 374 and 241-bp fragments, while the Gln allele was not digested (indicative of the absence of the *MspI* cutting site; Fig. 1).

DNA amplicons, as well as the digestion products, were electrophoresed through a 2–3% agarose gel (Genie, Bangalore, India) for resolution. The genotypes of >20% of the samples were reassessed in a double-blind manner by two independent researchers, to confirm the results. A random sample of 10% of each genotype was re-checked with sequencing to confirm the results.

### Statistical analysis

The observed frequencies of genotypes in CRC patients were compared with controls using the Chi-square test, or Fisher’s exact test when the expected frequencies were small. The Chi-square test was used to verify whether the genotype distributions were in Hardy-Weinberg equilibrium. P≤0.05 was considered to indicate a statistically significant result. Statistical analyses were performed using PASW version 18 software.

## Results

A total of 130 CRC cases and 150 control subjects were included in this study. The CRC patients consisted of 76 males and 54 females (M/F ratio, 1.41) and the control subjects consisted of 88 males and 72 females (M/F ratio, 1.2; data not shown). The mean age in both the patient and control groups was 53 years. No significant gender- or age-related differences were observed between the groups (P>0.05). Furthermore, out of 130 confirmed cases of CRC, 125 cases were sporadic, 4 were familial adenomatous polyposis and one case was hereditary non-polyposis (Lynch syndrome) CRC. All but one CRC case had adenocarcinoma and one had squamous cell carcinoma of the basal cell type. Fifty-two cases had carcinoma in the colon and 78 had carcinoma in the rectum; 78 were rural and 39 urban, and 81 were smokers and 49 non-smokers (Table II).

Among the CRC cases, we found the frequency of the *XRCC1* genotype to be 48.5% GG (63/130), 28.5% AG (37/130) and 23.0% AA (30/130), while the frequency in the general control population was 50.0% GG (75/150), 20.0% AG (30/150) and 30.0% AA (45/150). The overall association between the *XRCC1* polymorphism and the CRC cases was found to be non-significant (P>0.05; Table II). Furthermore, an independent analysis for the AG and AA genotypes revealed a significant correlation with the risk of CRC (P<0.05). The overall hazard ratio of the *XRCC1* A allele in CRC was 0.79 (95% CI, 0.44–1.4).

The correlation of the *XRCC1* polymorphic status with the clinicopathological characteristics was also carefully analyzed. We found a significant association (P<0.05) of the A allele with the age, gender, dwelling, tumor location, nodal status and tumor grade of the patients (P<0.05; Table II).

## Discussion

In this hospital-based case-control study of CRC patients in Kashmir, we analyzed the polymorphism of the *XRCC1 R399Q* gene, a DNA repair gene, and its concomitant role in modulating the risk of CRC.

The *XRCC1* gene belongs to a DNA repair gene family and encodes for a protein which plays a role in the repair of single-strand breaks (SSB) and in base excision repair (BER) (23). Shen *et al*(14) first of all reported the three common polymorphisms for the *XRCC1* gene occurring at its conserved sequences, all of which affect the coding region of the gene. These coding polymorphisms result in amino acid substitutions affecting the overall activity of the synthesized proteins, which are reported to affect codons 194 (Arg>Trp), 280 (Arg>His) and 399 (Arg>Gln) (23).

We found noteworthy results in our population comparable to those of Abdel-Rahman *et al*(15), since we found the frequency of the *XRCC1* genotype to be 48.5% GG (63/130), 28.5% AG (37/130) and 23.0% AA (30/130) in CRC cases. This frequency distribution is also comparable to that observed in the study of Wang *et al*(25).

In the case of the *XRCC1* Arg194Trp polymorphism, a few studies reported a reduced risk of cancer associated with the 194Trp variant form (16) while another study by Skjelbred *et al*(23) reported no association of the codon 194 polymorphism with the risk of CRC. In one of the studies, which involved the investigation of the *XRCC1* Arg280His allele and the risk of cancer, no association was observed (19). The *XRCC1* Arg399Gln polymorphism has been well studied in many cancers and positive correlations have been established; however, the results from these studies are not consistent (15,17,19–21,26).

The Arg399Gln polymorphism of the *XRCC1* gene resides at the C-terminal side of the poly (ADP-ribose) polymerase interacting domain which has been indicated as a protein-protein interaction module in many proteins involved in DNA repair mechanisms in the cell (27).

The majority of epidemiological case-control studies did not find any significant correlation between the *XRCC1* 399Gln variant and the risk of CRC (23,24,28,29), However, a case-control study carried out on a Taiwanese population found that an increased risk of CRC correlated with the *XRCC1* 399Arg/Arg genotype when compared with the *XRCC1* 399Gln in younger subjects (21). Although a study on a Norwegian population by Skjelbred *et al*(23) reported the *XRCC1* 399Gln allele to be related to a reduction in the incidence of high-risk adenomas, there was no association with any risk of carcinomas. In contrast to these reports, Abdel-Rahman *et al*(15) observed a significantly increased risk of CRC with the *XRCC1* 399Gln allele in their study on an Egyptian population and this effect was shown to be more significant among urban residents. In addition, Hong *et al*(19) demonstrated a positive association for this polymorphism in their study on a South Korean population.

Although our study on a Kashmiri population did not find any significant correlation between the *XRCC1* Arg399Gln polymorphism and an increased risk of CRC, similarly to many of the already reported studies, we did find an increased risk of CRC among Arg/Gln heterozygous cases compared to the controls (OR=1.47; 95% CI, 0.82–2.64). We also found a significant correlation between the 399Gln allele and the various clinicopathological parameters (Table II), particularly for gender (females), dwelling (rural) and tumor location (rectum). As already noted by Wang *et al*(25), differences due to gender may arise either due to physiologically different effects of the *XRCC1* 399Gln allele or due to different dietary habits, lifestyles and other genetic factors.

The interaction of the polymorphism with various environmental factors causes an increase in the overall susceptibility to CRC in any population (30,31). Therefore, we also hypothesize that since our population is exposed to certain environmental and dietary risks, including the consumption of sun-dried and smoked fish and meat, dried and pickled vegetables, red chilli, hakh (a leafy vegetable of the Brassica family), hot noon chai (salted tea) and hukka (water pipe) smoke (2,3,5), these may play a significant role in modulating the effect of the polymorphism in a dominant model of inheritance. As previously reported, the etiology and incidence of various gastrointestinal cancers in this population has been attributed to probable exposure to nitroso compounds, amines and nitrates, reported to be present in local foodstuffs, the majority of which have been shown to contain significant irritants and carcinogens (6).

The effect of 399Gln is also increasingly amplified in the carriers due to the impairment (or reduction) of the repair pathways, which in turn is reflected in significantly higher DNA adduct levels and an increased sister chromosome exchange frequency when compared with 399Arg (32).

We conclude that there is a significant correlation between the *XRCC1* A870G polymorphism and the risk of CRC in the ethnic Kashmiri population. These correlations now need to be authenticated in a large sample study, in order to discern racial differences and determine the aggressiveness of CRC.

## Figures and Tables

**Table I:** Genotype frequencies of the *XRCC1* gene polymorphism in CRC cases and controls.

*XRCC1* genotype	CRC cases (n=130)	Controls (n=150)	OR (95% CI); P¥; Fψ	χ^2^; P-value (overall)
GG (wild)	63 (48.5%)	75 (50.0%)	1	0.40; 0.81
AG (heterozygous)	37 (28.5%)	30 (20.0%)	1.47 (0.82–2.64); 0.19; 0.23	
AA (variant)	30 (23.0%)	45 (30.0%)	0.79 (0.44–1.4); 0.43; 0.47	

CRC, colorectal cancer; ¥, Pearson’s P-value; ψ, Fisher’s exact P-value.

**Table II:** Association between the *XRCC1* polymorphism and clinicopathological characteristics.

		No. of cases (n=130)^a^			
Variables	No. (%)	GG (n=63)	AG (n=37)	AA (n=30)	χ^2^; P-value
Age group					
≤50	48 (36.9)	14	20	14	**11.73; 0.002**
>50	82 (63.1)	49	17	16	
Gender					
Female	54 (41.54)	22	12	20	**10.20; 0.006**
Male	76 (58.46)	41	25	10	
Dwelling					
Rural	91 (70.0)	36	33	22	**11.61; 0.003**
Urban	39 (30.0)	27	4	8	
Smoking status					
Ever	81 (62.3)	41	23	17	0.61; 0.737
Never	49 (37.7)	22	14	13	
Tumor location					
Colon	52 (40.0)	17	22	13	**10.42; 0.005**
Rectum	78 (60.0)	46	15	17	
Nodal status					
Involved	88 (67.7)	50	18	20	**10.08; 0.006**
Not involved	42 (32.3)	13	19	10	
Tumor grade					
WD	98 (75.4)	48	28	22	**0.09; 0.956**
MD+PD	32 (24.6)	15	9	8	

a One was squamous cell carcinoma. Significant P-values are shown in bold. WD, well-differentiated; MD, moderately differentiated; PD, poorly differentiated.
